# From Byproduct to Platform: Can Kraft Lignin Outcompete Lignosulfonates?

**DOI:** 10.1021/acspolymersau.6c00111

**Published:** 2026-09-04

**Authors:** Jéssica S. Rodrigues, Amanda de S. M. de Freitas, Kumar D. Dutta, Mallika Saha, Leonardo F. Fraceto

**Affiliations:** † Institute of Science and Technology, São Paulo State University (UNESP), Av. Três de Março 511, 18087-180 Sorocaba, São Paulo, Brazil; ‡ Department of Finance and Banking, 221126 Patuakhali Science and Technology University, Dumki, Patuakhali 8602, Bangladesh; § Department of Accounting and Information Systems, University of Barishal, Kornokathi, Barishal 8254, Bangladesh

**Keywords:** lignosulfonates, lignin, dispersants, bioeconomy, techno-economic analysis, circular chemistry

## Abstract

Lignosulfonates (LS) have long set the standard of dispersants for construction, agrochemical, oilfield, and animal feed industries, due to their amphiphilic structure—aromatic domains linked with sulfonated functionalities, which result in efficient and cost-effective colloidal stabilization. Nevertheless, LS production is intrinsically limited by the falloff in sulfite pulping, which is today only a minor percentage of the world’s pulp manufacturing. On the other hand, kraft lignin (KL) is produced at multimillion-tonne levels annually and is now being acknowledged as a renewable aromatic polymer of strategic importance. Recent studies indicate that fractionation and controlled functionalization of KL can produce dispersants that match or outperform LS in selected systems, providing quantitative evidence that its chemical tunability can translate into competitive dispersant performance under defined application conditions. But these benefits come with problems of heterogeneity, process complexity, scalability, and cost. This Perspective argues that, owing to its chemical tunability, industrial availability, and potential for process innovation, KL should be regarded not as a universal replacement for LS, but as a platform capable of competing in next-generation dispersant systems where tailored performance justifies additional processing. By combining chemistry, technology limitations, and economics, lignin-based materials have proven to be important enablers of circular and low-carbon industrial technologies.

## Graphical abstract

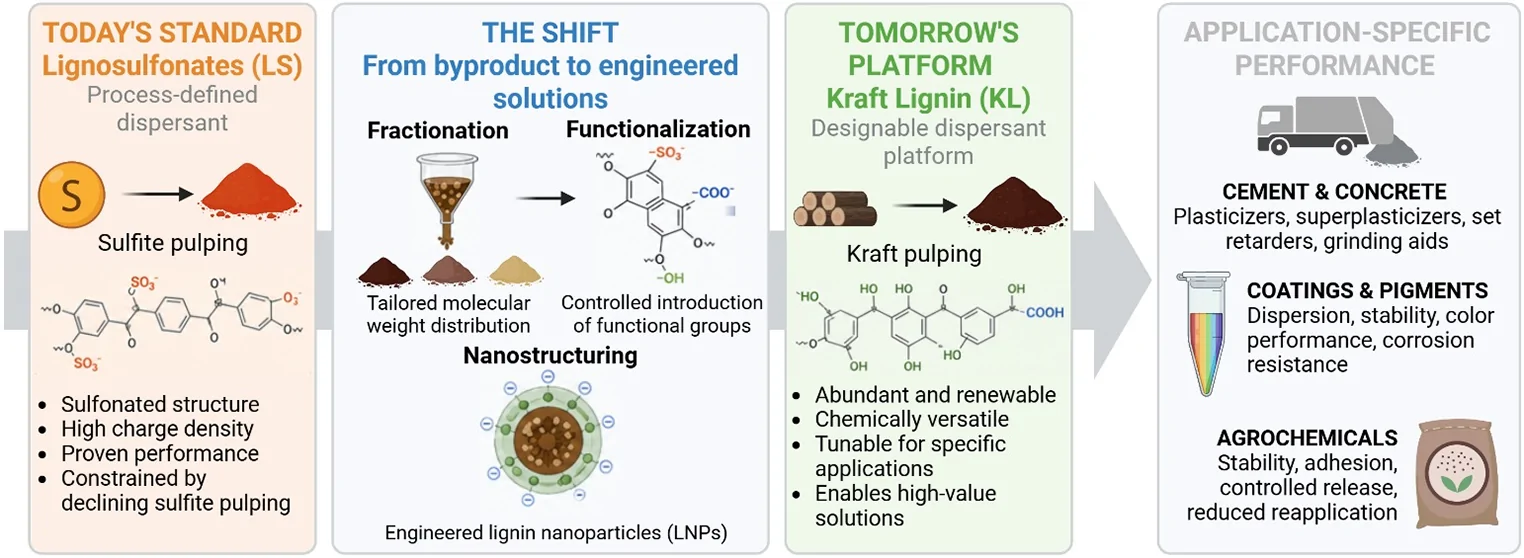

## 1 A Structural Shift in Dispersant Chemistry: A New Model for the Dispersant Industry

With decades of reliable dispersion efficiency and cost-effective performance in concrete,^1^ coatings,^2^ agriculture,^2^ and energy,^3^ lignosulfonates (LS) have shown great potential for global commercial applications and industry.^3^ Characterized by an aromatic backbone and sulfonate functionalities, the amphiphilic nature has long been used as a model for colloidal stabilization.^3^ Still, the industrial rationale for this model is being dramatically reconfigured. Kraft pulping now dominates global chemical pulp production and has largely displaced sulfite pulping, the process from which LS are generated as water-soluble byproducts. Consequently, the modern pulp industry produces substantially larger upstream quantities of kraft lignin (KL) than lignosulfonates.^3^ Global chemical wood pulp production reaches hundreds of millions of tonnes annually, illustrating the scale and consolidation of the modern pulping industry.^4^

This shift illustrates an essential paradox in the bioeconomy: the demand for renewable, inexpensive dispersants remains on the rise,^5,6^ while the dominant biobased origin is becoming more limited. Under this scenario, KL emerges as an abundant and chemically versatile platform whose derivatives can match or outperform LS in selected applications when properties such as charge density, molecular-weight distribution, and amphiphilic balance are appropriately tailored.^3^ Lignin is produced in large quantities in contemporary pulping plants but has a versatile and variable chemical structure that can be engineered for greater performance. Improvements in fractionation, targeted modification, and nanoparticle engineering are driving not only substitutions, but also performance optimization beyond traditional systems. Together, these product trends suggest that this transition is not just a material replacement, but also a technological shift in dispersant chemistry. This imbalance is not confined to market dynamics; it reflects a fundamental geographical and industrial asymmetry: diminishing sulfite-based supply chains and increasing kraft pulping capacity. LS output is limited by the decreasing sulfite pulping base; KL is produced in the prevailing kraft pulping industry. This establishes a basic mismatch between limited availability and expanding upstream potential.

According to FAO forestry statistics^4^ and its recent lignin market analyses, commercial lignosulfonate production remains concentrated in regions historically associated with sulfite pulping particularly North America and Northern Europe. For example, Norway, Sweden, Germany, Canada and the United States. Meanwhile, KL availability is closely in line with the dominant kraft pulp industry. Major production hubs are at countries such as the United States, Canada, Brazil, China, Finland and Indonesia, as illustrated in Figure 1. But, despite high abundance and chemical potential, competing with LS would need more than that it will need to overcome technological, economic, and market barriers keeping LS from making more widespread adoption happen.

**Figure 1:**
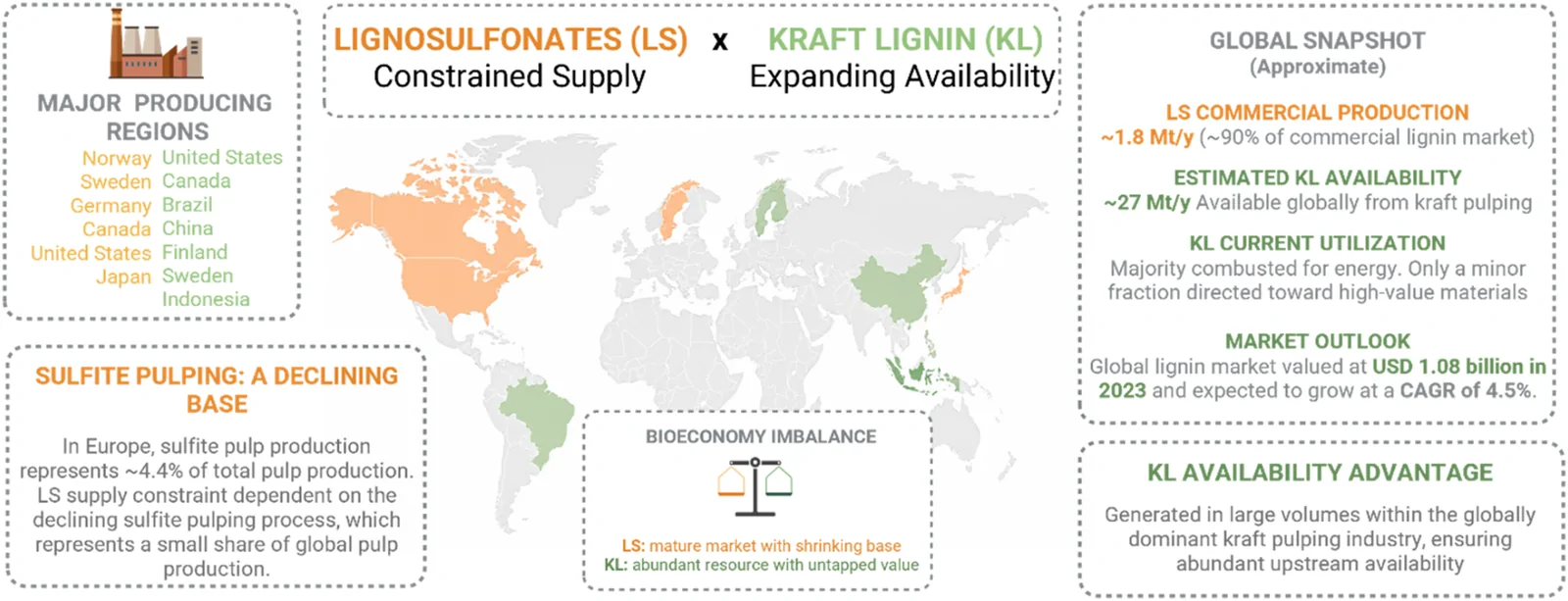
Global comparison of lignosulfonate (LS) supply and kraft lignin (KL) availability. Commercial LS production (∼1.8 Mt·y^–1^) is associated with the declining sulfite pulping sector, which represents approximately 4.4% of total pulp production in Europe, resulting in a relatively constrained and geographically concentrated supply. In contrast, KL is generated within the globally dominant kraft pulping industry, providing an estimated upstream availability of ∼70 Mt·y^–1^, although only a minor fraction is currently directed toward high-value applications. The map highlights major LS- and KL-producing regions and illustrates the resulting bioeconomy imbalance between a mature LS market with a constrained production base and an abundant but underutilized KL resource. Global indicators provide additional context on current utilization and market outlook.^3,5,7–9^ Created with BioRender.

## 2 The Chemical Perspective: Pre-Functionalized Dispersants to Designable Platforms

LS have historically set the benchmark for biobased dispersants because of their intrinsic amphiphilic architecture, combining an aromatic backbone with sulfonate functionalities.^10^ Intermediary effect between interfacial adsorption and electrostatic stabilization is responsible for the dispersant performance at the colloidal level.^7^ Aromatic domains attach to hydrophobic surfaces by π–π interactions and van der Waals forces, while sulfonate groups (−SO_3^–^_) give a strong negative charge, which contributes to electrostatic repulsion and reduces aggregation.^11^ This dual mechanism forms the basis for dispersion efficiency in many industrial systems, from cement suspensions to agrochemical formulations and mineral slurries. Figure 2 conceptually summarizes the fundamental differences between the process-defined functionality of LS and the designable chemical platform offered by KL.

**Figure 2:**
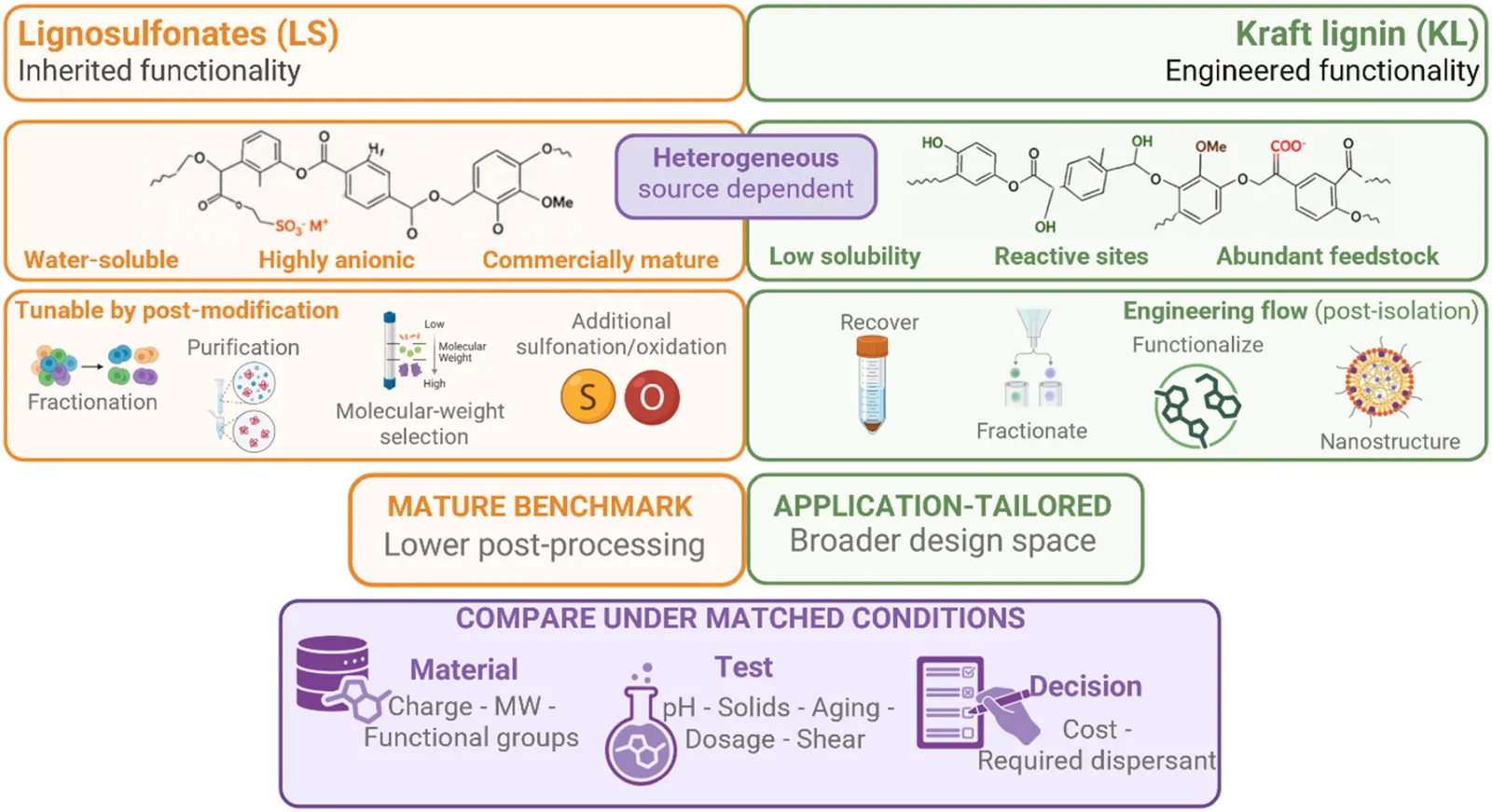
Conceptual comparison of lignosulfonates (LS) and kraft lignin (KL) as platforms for dispersant development. LS are heterogeneous and polydisperse lignin-derived polyelectrolytes whose anionic functionality is largely introduced during sulfite pulping, although their properties can be further adjusted through purification, fractionation, and postmodification. KL is likewise heterogeneous and polydisperse but generally requires recovery, specification control, fractionation, targeted functionalization, and/or nanostructuring to achieve application-relevant water compatibility, charge density, and amphiphilic balance. The structures shown are representative motifs and do not correspond to unique or fully resolved molecular structures. The competitiveness of LS- and KL-derived dispersants should be evaluated under matched application conditions, including dosage, pH, ionic strength, solids loading, substrate, aging time, and temperature, and should ultimately be assessed in terms of delivered cost per unit of required dispersant performance. Created with BioRender.

However, this functionality is intrinsically determined by sulfite pulping chemistry. Sulfonation is carried out in an acid environment of lignin solubilization resulting in a dispersion of the anionic moieties in the partly depolymerized structure.^12^ Therefore, LS shows a high variability of molar mass and charge density which also influence adsorption mechanism, interfacial pattern, and sensitivity to ionic strength and pH. Such process-induced structure–property relationship tailoring and optimization for applications is severely constrained.

Conversely, the KL is a chemically versatile platform allowing for deliberate molecular designs.^13^ KL keeps the high proportion of reactive functional groups such as phenolic hydroxyl (Ar–OH), aliphatic hydroxyl (R–OH) and carboxylic (−COOH),^13^ all of which have multiple sites for selective reformation.^14–16^ As a result, critical physicochemical parameters - such as charge density, polarity, hydrophilic–lipophilic balance - can be adjusted according to particular interfacial needs and move beyond the fixed functionality of LS.^13^ In this respect LS may be seen as predetermined dispersants, whereas KL acts as an engineering platform whose interfaces can be deliberately created. Anionic groups (−SO_3^–^_) introduced during sulfonation, comparable to those found in LS, are present here; oxidative routes (like TEMPO-mediated or alkaline oxidation) make carboxylate functionalities (−COO^–^)^17^ that enhance hydrophilicity and permit pH-responsive behavior.^18^ Further modifications like esterification or acylation would expand these parameters further by providing more control over amphiphilicity and adsorption affinity.^13^ As such, lignin-derived dispersants can be systematically tailored for the desired effect with tailored performance.

In addition to chemical engineering, advancements in lignin fractionation to further improve KL treatment have permitted the molar mass distribution of KL fractions to narrow and functional group content to be more manageable.^13^ These techniques reduce polydispersity and batch-to-batch variation, allowing improved reproducibility and much more stable interfacial response. This refinement was essential for the dispersant use, and molar mass and functional group distribution determine adsorption kinetics and steric stability directly. Recent studies by Rodrigues et al.^19^ showed that the structured form of the fractionated KL into an aqueous acetic acid is derived from structural and morphologically separate fractions with modified molecular structure and improved colloidal behavior, showing the influence of structure with interfacial activity

Nanostructuration introduces an additional level of control. Lignin nanoparticles (LNP), obtained via antisolvent precipitation, microemulsion templating, or spray-drying, exhibit defined size distributions and tunable surface charge.^20^ An interface-like core–shell-like structure in which hydrophobic domains are located within the core and functional groups are exposed at the surface promotes interfacial activity and allows electrostatic and steric stabilization. Consequently, LNP provide an additional route to control particle size, surface charge, and interfacial behavior, potentially improving electrostatic and steric stabilization. However, direct performance comparisons with conventional LS remain application-dependent and should be evaluated under matched conditions of pH, ionic strength, dispersant dosage, and solids loading.

Because LS and KL are heterogeneous technical materials, a single, unique molecular formula cannot represent their chemical structures. The structures shown in Figure 2 therefore correspond to representative structural motifs used to illustrate their dominant functional features. In LS, the aromatic lignin backbone is shown with representative sulfonate groups introduced during sulfite pulping. In KL, the aromatic backbone is shown with phenolic hydroxyl, aliphatic hydroxyl, and carboxylic groups that can serve as sites for controlled functionalization.

This transition from process-defined to molecular and supramolecular functionality represents a fundamental shift in the design of lignin-based dispersants. While LS functionality is largely determined by sulfite pulping, KL provides a broader design space in which functional groups and molecular-weight distribution can be tailored through fractionation, oxidation, sulfonation, esterification, and nanostructuring.

### 2.1 Quantitative Evidence for Application-Specific Competitiveness

Several application-specific case studies support the transition from conceptual tunability to demonstrated performance (Table 1). Hydrogen-peroxide oxidation of KL produced an oxidized lignin containing 1.53 mequiv g^–1^ of carboxylate groups. When applied at 40 mg L^–1^ to a 4 g L^–1^ kaolin suspension at pH 5, the oxidized KL produced a relative turbidity of 1.18 and outperformed a commercial LS under the conditions evaluated.^21^ This result provides a direct example of a KL-derived dispersant exceeding LS performance in a defined mineral-suspension system.

**Table 1:** Quantitative Evidence of the Application-Specific Performance of KL-Derived Dispersants^a^

KL-derived system	application/test conditions	quantitative performance	benchmark/interpretation	refs
H_2_O_2_-oxidized KL (OKL)	Kaolin; 4 g·L^–1^; pH 5; 40 mg·L^–1^ OKL	Carboxylate: 1.53 mequiv g^–1^; relative turbidity: 1.18	**Direct comparison:** outperformed commercial LS under the tested conditions	^21^
Carboxymethylated KL (CL-1)	Coal–water slurry; 50 wt % solids; 0.05–0.25 wt % dispersant	Instability index <0.6 at 0.10 wt %; clarification: 3.385 → 2.77%T h^–1^; sedimentation: 0.308 → 0.242 mm h^–1^; ζ: – 34.6 → – 52 mV; contact angle: 45.3° → 34.6°	**Indirect comparison:** improved stability relative to untreated slurry; no direct LS benchmark	^22^
Sulfomethylated/sulfobutylated KL	Kaolin suspension	Sulfonate: KL 0.02; SML 1.88; SBL 1.89 mmol g^–1^	Effective at neutral pH; performance decreased outside this range	^23^
Fractionated + esterified KL	Oil-related formulations	Best dispersing behavior reported for higher-MW fraction esterified with succinic anhydride	Compared with commercial LS; demonstrates contribution of MW and functionalization	^24^

a Direct and indirect comparisons are distinguished because experimental conditions and performance metrics differ among studies.

In concentrated coal–water slurries containing 50 wt % solids, carboxymethylated softwood KL (CL-1) also produced measurable improvements in colloidal stability. At a dosage of 0.05 wt %, the clarification and sedimentation rates decreased from 3.385 to 2.77%T h^–1^ and from 0.308 to 0.242 mm h^–1^, respectively. Increasing the CL-1 dosage to 0.10 wt % reduced the instability index to below 0.6.^22^ The same treatment shifted the zeta potential from −34.6 to −52 mV and reduced the coal-surface contact angle from 45.3° to 34.6°, indicating enhanced electrostatic repulsion and wettability.^22^ These results should be interpreted as an improvement relative to the untreated slurry because the study did not include a direct LS benchmark under the same experimental conditions.

Chemical sulfonation provides a further example of application-specific tuning. Sulfomethylation and sulfobutylation increased the sulfonate content of KL from 0.02 mmol g^–1^ to 1.88 and 1.89 mmol g^–1^, respectively. Both derivatives were effective in kaolin suspensions at neutral pH, whereas their performance decreased under acidic and alkaline conditions.^23^ This pH dependence illustrates that functionalization can improve water compatibility and interfacial stabilization while also introducing an application-specific operating window.

Fractionation combined with esterification has likewise generated KL derivatives with significant dispersing properties relative to a commercial LS in oil-related formulations. The most favorable dispersing behavior was reported for a higher-molecular-weight fraction esterified with succinic anhydride.^24^ Although the studies summarized here use different substrates, formulations, dosages, and response variables, they collectively demonstrate that the competitiveness of KL is not an intrinsic property of the unmodified polymer. Rather, it emerges from the deliberate control of charge density, molecular-weight distribution, molecular architecture, and interfacial affinity.

The evidence should nevertheless be interpreted with caution. The available studies differ substantially in substrate, solids concentration, pH, dispersant dosage, aging time, and performance metric, preventing a universal quantitative ranking of KL and LS. The evidence supports a more specific conclusion: engineered KL can match or outperform LS in selected application conditions, whereas standardized head-to-head comparisons with commercial LS remain necessary to define the technological boundaries of this competitiveness.

Overall, the performance of KL-based dispersants is governed not only by chemical composition but also by the interplay among molecular architecture, functional-group distribution, surface chemistry, and supramolecular organization. The quantitative evidence summarized above demonstrates that this tunability can translate into measurable improvements in dispersion performance under defined conditions. However, converting this chemical versatility into robust industrial technologies will ultimately depend on reproducibility, scalable processing, standardized performance evaluation, and cost-effective implementation.

## 3 The Economic Perspective

The economic comparison between LS and KL should distinguish market incumbency from resource potential. LS benefits from process-integrated sulfonation and mature dispersant supply chains, whereas KL has a much larger upstream resource base but remains only partly commercialized. Global commercial KL production capacity was estimated at approximately 112 kt yr^–1^ in 2023,^25^ while the technically recoverable potential from kraft mills has been estimated at about 27 Mt yr^–1^ assuming a 40% lignin outtake.^26^ The disparity highlights substantial unrealized supply potential, as current commercial capacity represents only a small fraction of the technically recoverable resource. However, converting this resource potential into a competitive market supply depends on recovery, purification, process integration, and downstream upgrading costs.

Recent techno-economic assessments of KL-derived products show that economic performance is highly sensitive to process integration, scale, and reagent or solvent recovery. For example, Forssell et al.^27^ estimated minimum selling prices of €0.68 kg^–1^ for an integrated epoxidized-KL plant and €0.86 kg^–1^ for a standalone configuration; solvent recycling was the dominant sensitivity parameter, with above 99% recycling achieved in the modeled process. Similarly, Ahire et al.^28^ found that the modeled production cost of a KL-containing bioadhesive declined from US$1.57 to US$1.19 kg^–1^ as plant scale increased from 10 to 50 MT per batch. Although these products and process boundaries differ from those of dispersants, they illustrate that the economics of KL-derived materials cannot be inferred from feedstock or recovery cost alone.

The same distinction is central to the comparison between ultrafiltration (UF) and commercial KL recovery technologies. Jönsson and Wallberg^29^ estimated UF recovery costs of approximately €60 t^–1^ for cooking liquor and €33 t^–1^ for black liquor under the principal configurations evaluated, with costs approaching €17 t^–1^ under a lower-volume-reduction cooking-liquor case. These estimates concern membrane separation rather than production of a purified, specification-compliant KL product; further concentration and purification add costs, alongside membrane replacement, cleaning, energy, and capital requirements. By contrast, LignoBoost and LignoForce were developed as integrated lignin–outtake processes. LignoBoost has achieved commercial implementation and uses sequential acidification, filtration, and washing to reduce ash below approximately 1 wt %, whereas LignoForce introduces black-liquor oxidation before acidification to alter particle formation, simplify filtration, and reduce H_2_S emissions.^26^ These features help explain why precipitation-based systems have achieved commercial deployment despite favorable historical UF separation-cost estimates. A head-to-head techno-economic assessment using common system boundaries is therefore still needed.

For KL-based dispersants, the relevant economic benchmark is consequently the delivered cost per unit of application-relevant performance, rather than the cost per ton of recovered lignin. Recovery, purification, drying, fractionation or functionalization, reagent recycling, dosage, and product consistency must therefore be considered together. Current evidence does not support a general claim that KL will undercut LS on absolute cost; rather, KL is most likely to compete where tailored functionality, supply security, or lower effective dosage provides sufficient value to offset the additional processing required.

## 4 Forward-Looking View

The central industrial challenge is shifting from recovering KL to controlling its specification. Feedstock and pulping conditions alter molecular-weight distribution, functional-group density, ash, sulfur, and reactivity, limiting reproducibility across downstream applications. Fractionation, selective solvation, and controlled precipitation can narrow material variability, while standardized characterization can define and verify application-specific KL grades.^26,30,31^ The objective should not be maximal chemical modification, but the minimum processing intensity required to reach a reproducible performance target.

Recent scale-up work illustrates a practical route. Ghosh et al.^32^ transferred aqueous-ethanol KL fractionation to a 250-L reactor using 17 kg of dry lignin. The soluble fraction was used in biobased alkyd coatings, while the insoluble fraction was subsequently oxidized and evaluated as a carbon-black dispersant against Vanillex, a commercial modified lignosulfonate. This cascading use of both fractions is industrially relevant because it couples specification control with coproduct valorization rather than treating fractionation as a purification penalty alone. The study, however, remains pilot-scale and does not establish commercial cost parity with LS. In parallel, the economic advantage of mill integration and near-complete solvent recycling demonstrated by Forssell et al.^27^ indicates that process design will be as important as molecular design during scale-up.

The next evidence threshold should therefore be pilot- and demonstration-scale production of dispersant-grade KL under matched application conditions. Studies should report modification yield, solvent and energy inputs, solvent recovery, batch-to-batch variability, continuous operating stability, product specifications, and performance against commercial LS at equivalent dosage, pH, ionic strength, and solids loading. Such data would permit a genuine cost-performance comparison rather than extrapolation from laboratory functionality.

KL is thus unlikely to displace LS through simple commodity substitution. A more credible pathway is application-specific competition: standardized KL grades, integrated recovery and modification, efficient reagent recycling, and validated application-specific performance. The decisive question is not whether KL is cheaper to recover, but whether it can deliver a lower cost per unit of required dispersant performance at industrial scale.

## 5 Conclusion

KL represents one of the most abundant yet underutilized renewable aromatic resources available today, offering a broader design space than the largely process-defined functionality of conventional LS. However, this versatility comes at a cost. Unlike LS, where functionality is embedded within the production process, KL requires deliberate engineering, introducing challenges related to reproducibility, scalability, and process integration. As a result, the transition from LS to KL will not occur through direct substitution, but through selective adoption in applications where performance justifies complexity. The central challenge is therefore not to demonstrate lignin’s potential, but to operationalize it. The future of lignin-based dispersants will ultimately be defined not by feedstock availability, but by the ability to translate chemical tunability into scalable, reliable, and economically viable technologies.
